# Case Report: Mature Plasmacytoid Dendritic Cell Proliferation Associated With a Lymphoid Neoplasm

**DOI:** 10.3389/fonc.2022.903113

**Published:** 2022-07-06

**Authors:** Fei Fei, Michaela Liedtke, Oscar Silva

**Affiliations:** ^1^ Department of Pathology, Stanford University School of Medicine, Stanford, CA, United States; ^2^ Division of Hematology, Stanford University School of Medicine, Stanford, CA, United States

**Keywords:** mature, BPDCN, pDC proliferation, MPDCP, lymphoid, T-ALL, leukemia

## Abstract

Mature plasmacytoid dendritic cell proliferations (MPDCPs) are clonal, non-malignant pDC proliferations that have been reported to occur in association with myeloid neoplasms such as CMML, AML (pDC-AML), and, rarely, MDS or MPNs. Here we report the first case of a MPDCP associated with T-lymphoblastic leukemia (T-ALL), a lymphoid neoplasm. The MPDCP in this case involved ~50% of the bone marrow, was found in nodular aggregates, expressed CD123, CD4, and CD303, and lacked CD56 and TCL1 expression. In addition, the MPDCP lacked CD34 and TdT but showed aberrant expression of CD7, CD5, CD10, and CD13, markers expressed by the abnormal T-lymphoblastic cells. Mutational analysis demonstrated mutations in JAK3, NOTCH1, NRAS, KRAS, DNMT3A, and SH2B3 but no mutations in TET2, ASLX1 or ZRSR2. Analysis of the pDC frequency in a separate cohort of T-ALL and control patients demonstrated that bone marrow pDCs are often decreased in patients with T-ALL compared to controls. This is the first report of a MPDCP associated with a lymphoid neoplasm and provides further support that MPDCP can arise from a multipotent hematopoietic progenitor with lymphoid and dendritic cell potential.

## Introduction

Plasmacytoid dendritic cells (pDCs) are specialized immune cells that induce high levels of type I interferons in response to viral infections (1). Although pDCs are often a small fraction of the cellular composition within peripheral blood, bone marrow, and lymph node, in certain disease states, they may proliferate. The proliferation of pDCs can be broadly characterized as non-clonal:reactive, clonal:non-malignant, and clonal:malignant. The non-clonal:reactive proliferation of pDCs occurs in Kikucki–Fujimoto lymphadenopathy and systemic lupus erythematosus, whereas the clonal:malignant proliferation of pDCs is known as blastic plasmacytoid dendritic cell neoplasm (BPDCN) (2, 3). The less characterized of these pDC proliferations are the clonal:non-malignant proliferations currently known as mature pDC proliferations or pDC proliferations associated with myeloid neoplasms (4).

Mature plasmacytoid dendritic cell proliferations (MPDCPs) are morphologically mature pDC proliferations with a non-malignant clinical course characterized by expression of pDC markers (CD123, CD303, TCF4, etc.), low Ki67 proliferation index (<10%), and decreased to negative expression of CD56 (4). These proliferations may show aberrant loss of pDC markers or expression of CD34 and terminal deoxynucleotidyl transferase (TdT) (5, 6). MPDCPs are rare and have only been associated with myeloid neoplasms, suggesting that they arise from a clonally related myeloid-progenitor. The most common myeloid neoplasms associated with MPDCPs are acute myeloid leukemia (pDC-AML), particularly AML with *RUNX1* mutations, chronic myelomonocytic leukemia (CMML), and, rarely, myelodysplastic syndrome (MDS) or myeloproliferative neoplasms (MPNs) (5–9). Here, we describe the first report of a lymphoid neoplasm, T-lymphoblastic leukemia (T-ALL), occurring with a MPDCP that shows immunophenotypic similarities to the associated lymphoid neoplasm. We further interrogate the frequency of pDCs in the bone marrow of an additional cohort of patients with T-ALL compared with normal patients.

## Case Description

**Figure 1:**
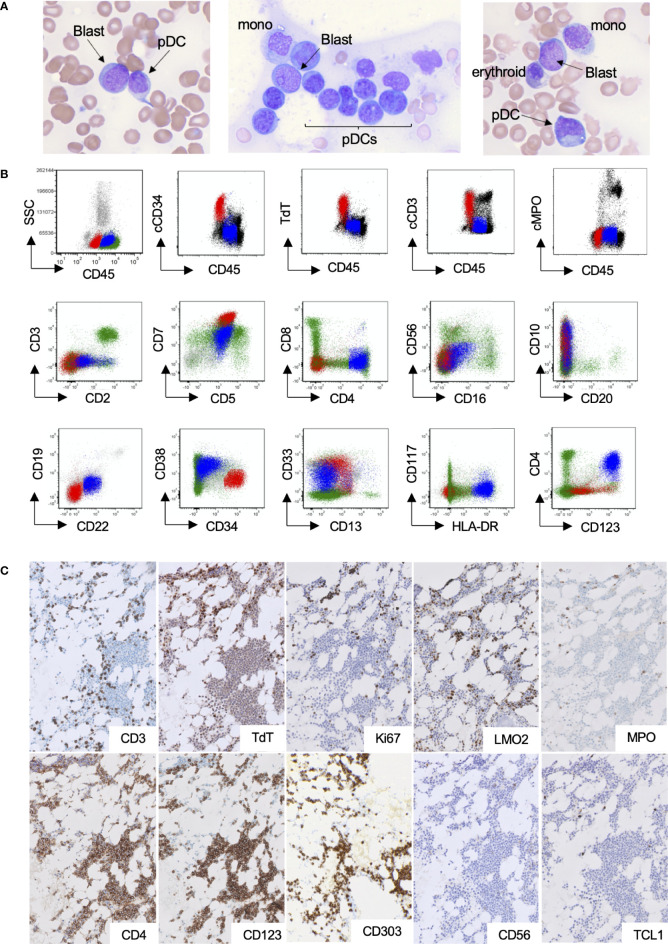
pDC proliferation within the bone marrow of a patient with T-ALL. **(A–C)** Representative bone marrow Wright–Giemsa aspirates (×600 magnification), demonstrating blasts, morphologically mature plasmacytoid dendritic cells, and other background hematopoietic cells. **(B)** Flow cytometry highlighting the abnormal T-lymphoblastic (red) and pDC populations (blue). **(C)** Immunohistochemical stains (×200 magnification), highlighting small clusters of T lymphoblasts (upper panel) and large aggregates of pDCs (lower panel).

A 51-year-old woman with a history of fibromuscular dysplasia and non–ST-elevation myocardial infarction presented with pancytopenia on complete blood count (white blood cell: 1.8 × 10^9^/L; hemoglobin: 10.8 g/dL; platelets: 160 × 10^9^/L; absolute neutrophil count: 0.7 × 10^9^/L; absolute lymphocyte count: 0.9 × 10^9^/L). Peripheral blood smear demonstrated atypical lymphoid cells concerning for blasts. A subsequent bone marrow aspiration and biopsy demonstrated decreased trilineage hematopoiesis with no morphological evidence of dysplasia to suggest MDS; however, an increase in blasts and atypical plasmacytoid cells were present in a background of other hematopoietic cells (**Figure 1A**). The atypical plasmacytoid cells were small to medium in size with irregular round-to-oval nuclei showing occasional eccentricity, mature chromatin, and basophilic cytoplasm. On the basis of a 200-cell manual differential of the bone marrow aspirate, the frequency of the different cell types was as follows: blasts, 24%; myelocytes, 1%; metamyelocytes, 0.5%; band neutrophils, 1%; erythroids, 14.5%; lymphocytes, 10.5%; plasma cells, 2%; monocytes, 1%; mast cells, 0.5%; atypical plasmacytoid cells, 45%. By flow cytometry, the blasts were abnormal T lymphoblasts and expressed cytoplasmic CD3, TdT, CD34, CD1a(subset), CD10(partial), CD5, CD7(bright), CD13(partial), CD33(partial), CD123(dim partial), and CD45(dim) and lacked expression of other lymphoid and myeloid markers including cytoplasmic MPO (**Figure 1B**, red, and **Supplemental Figure 1**). By flow cytometry, the atypical plasmacytoid cells were consistent with pDCs and expressed CD123(bright), CD4, HLA-DR(bright), CD33(moderate), CD7(moderate), CD5(moderate), CD2(dim), CD10(partial), CD1a(subset), CD22(dim), and CD45(moderate) and lacked CD34, TdT, CD56, cCD3, cMPO, monocytic markers, and other T-cell/B-cell markers (**Figure 1B**, blue, and **Supplemental Figure 2**). To visualize the distribution of T lymphoblasts and pDCs within the bone marrow, immunohistochemistry on serial sections of the core biopsy was performed. T lymphoblasts were seen as clusters of either CD3, TdT (dark brown nuclear staining), LM02, or Ki67-positive cells throughout the bone marrow (**Figure 1C**, upper panel). No clustering of MPO or lysozyme was identified. The mature pDCs constituted approximately 50% of the total bone marrow cellularity and were distributed predominantly as nodules/aggregates. The pDCs expressed CD4, CD123, CD303, and low Ki67 (<5%) and lacked TdT, CD56, and TCL-1 by immunohistochemistry (**Figure 1C**, lower panel). Of note, TdT showed low-level background staining in non-blasts, which was similar to that seen in medullary thymocytes which are known to be TdT-negative (**Supplemental Figure 3**). Overall, the morphologic and immunophenotypic features were concerning for T-ALL with an associated MPDCP.

To further characterize this T-ALL with associated MPDCP, cytogenetics and molecular studies were performed. Conventional cytogenetics showed a normal female karyotype and MDS fluorescence *in situ* hybridization for deletion 5q, 7q, and 20q, and trisomy 8 was negative. T-cell clonality studies by next-generation sequencing (NGS) demonstrated a clonal T-cell receptor beta chain (TRB) gene rearrangement. Targeted NGS mutational analysis using the Stanford Actionable Mutation Panel for Hematopoietic and Lymphoid Malignancies (HEME-STAMP) demonstrated the following mutations: DNMT3A p.S786* (31% VAF), SH2B3 p.R308* (26% VAF), JAK3 p.L857P (8% VAF), NOTCH1 p.L1678Q (4% VAF), NRAS p.G12V (4% VAF), and KRAS p.G12D (2% VAF). No mutations in TET2, ASLX1, or ZRSR2 were identified. These findings supported the diagnosis of T-ALL with an associated MPDCP and provided no definite evidence for BPDCN.

Positron emission tomography–computed tomography demonstrated hypermetabolic lymphadenopathy above and below the diaphragm. The patient was subsequently induced with cyclophosphamide, vincristine, doxorubicin, and dexamethasone.

## Methods

To determine whether increased pDCs are associated with T-ALL within the bone marrow, as has been recently demonstrated in a subset of AML cases, currently referred to as pDC-AML (5, 6), we identified patients with T-ALL and normal controls from our pathology database between January 2020 and December 2021 and assessed the frequency of bone marrow pDCs at diagnosis by flow cytometry—identified by their characteristic immunophenotype of CD45(moderate), CD123(bright), and HLA-DR(bright) surface expression. In addition, clinical, pathologic, and molecular data were collected. Statistical analysis was performed using GraphPad Prism. This study was approved by the institutional review board of Stanford University School of Medicine.

## Results and Discussion

**Figure 2:**
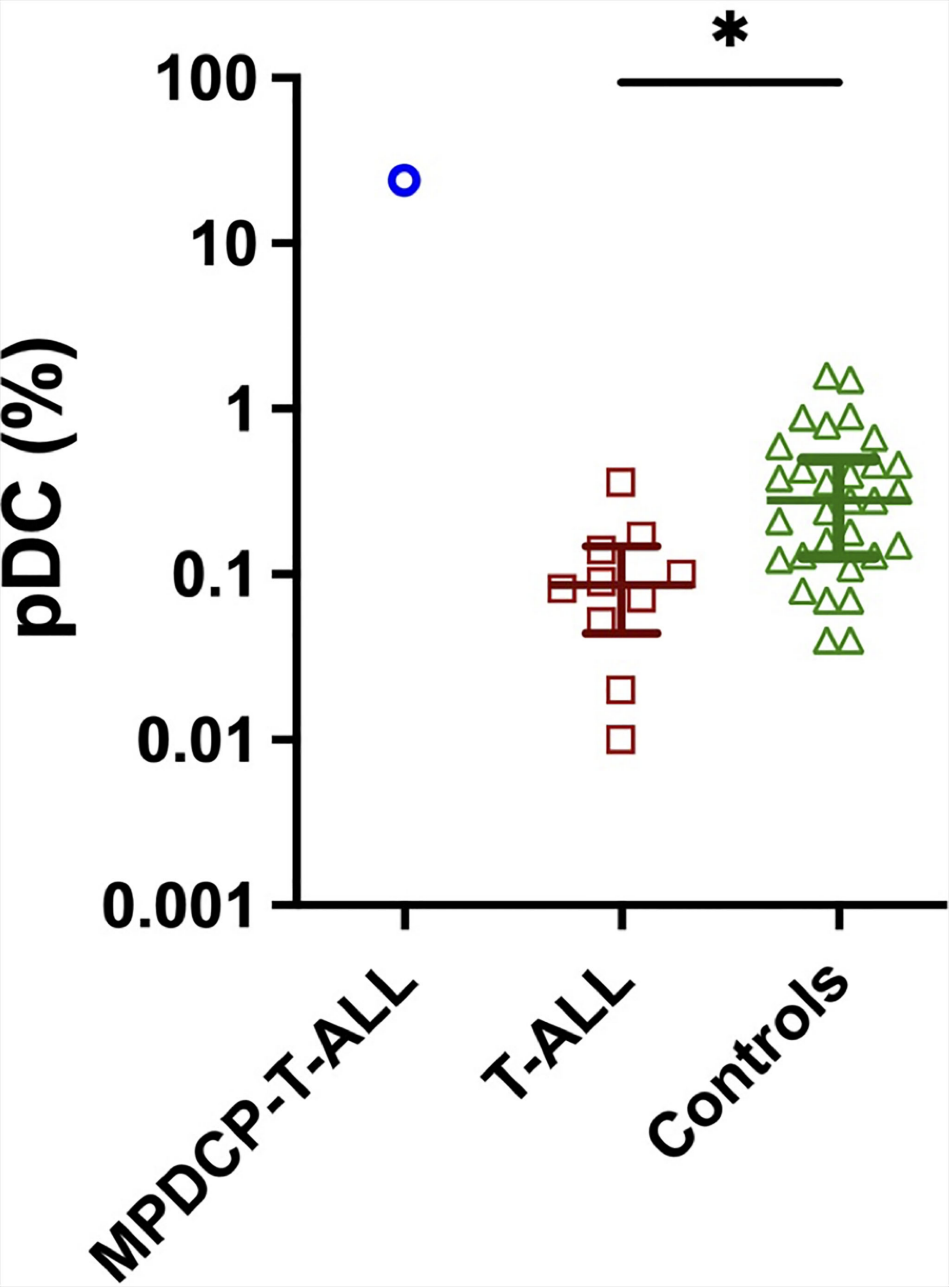
Decreased pDC are frequent in patients with T-ALL. pDC frequency (percentage of white blood cells) in bone marrow aspirates from our index case of MPDCP-T-ALL, and from patients with T-ALL (n = 10) and controls (n = 30). Presents are median ± interquartile range. *p < 0.05 based on unpaired t-test.

The median proportion of pDCs in normal bone marrow controls (n = 30) was 0.26% [interquartile range (IQR), 0.13%–0.46%], whereas, in patients with T-ALL (n = 10, not including the current patient), the median proportion of pDC was depleted at 0.086% (IQR, 0.05–0.14%) (**Figure 2**). Although additional cases of T-ALL with increased pDCs were not found, this cohort was small and thus further studies are required to determine if increased pDCs are a common finding in a subset of T-ALL, and if so, if there are unique clinicopathological features among these T-ALL cases.

The understanding of dendritic cell ontology, in particular pDC ontology, has recently been challenged with single-cell RNA sequencing (scRNAseq) studies. Prior to scRNAseq, there was evidence suggesting that pDCs derived from myeloid-derived precursors that additionally gave rise to conventional DCs (cDCs). However, recent studies have demonstrated that pDCs may additionally arise from Ly6D^+^ lymphoid progenitors which are distinct from the myeloid DC lineage (10, 11). These studies open the possibility of a shared precursor for both the pDC and T-lymphoblastic populations in our case. In addition, it prompts investigation of the clonal origin of rare cases of BPDCN occurring concurrently with T-ALL or peripheral T-cell lymphoma, in addition to BPDCNs aberrantly expressing cytoplasmic CD3 (12–15).

Although recent studies have provided evidence of a common shared precursor for pDCs and lymphocytes, the pathogenic mutations that may participate in shared clonal proliferations of pDCs and T-lymphocytes is unclear. In the present case, we identified pathogenic loss-of-function mutations in DNMT3A and SH2B3 at variant allele frequencies (VAFs) greater than those for JAK3 and NOTCH1, suggesting that DNMT3A and SH2B3 mutations may be initial, founding mutations in a shared hematopoietic precursor that can give rise to sub-clonal populations of pDCs and T lymphoblasts. Indeed, identical DNMT3A mutations with the same VAF have been identified in sorted blasts and pDCs of pDC-AML (5, 6), suggesting that dysregulation of epigenetic modifiers may be an early molecular event in the progression of clonal pDC proliferations associated with a subset of hematological neoplasms.

In summary, we present the first case of a MPDCP associated with T-ALL, a lymphoid neoplasm. We propose that the MPDCP seen in this case should be considered a “pDC proliferation associated with a *hematological* neoplasm” given the extensive proliferation of pDCs in nodular aggregates, lack of CD56 and TCL1, and the co-expression of lymphoid antigen also expressed by the abnormal T-lymphoblastic cells. A limitation of this case report is that sorting of pDCs and T lymphoblasts was not performed to determine which mutations are mutually expressed by both pDCs and T lymphoblasts; however, the aberrant cross-lineage antigen expression (CD5, CD7, CD10, CD1a, and CD13) on both blasts and pDCs supports the idea of a shared clonal origin between these populations. However, we cannot exclude the possibility that the MPDCP in this case developed from a clonally distinct hematopoietic precursor independent of the T-ALL. Future prospective studies should aid to definitely establish mutational clonality between MPDCPs and lymphoid neoplasms, as has recently been done in myeloid neoplasms (7). In addition, the clinical significance of an associated MPDCP in lymphoid neoplasms is currently unknown; however, our patient was noted to have persistent disease after 30 days of induction chemotherapy.

## Data Availability Statement

The original contributions presented in the study are included in the article/**Supplementary Material**, further inquiries can be directed to the corresponding author/s.

## Ethics Statement

This study was reviewed and approved by the Stanford IRB, Protocol 63202. Informed consent was obtained from the individual for the publication of any potentially identifiable images or data included in this article.

## Author Contributions

FF and OS designed the study, analyzed the data, and wrote the manuscript. ML provided clinical insight and edited the manuscript. All authors contributed to the article and approved the submitted version.

## Conflict of Interest

The authors declare that the research was conducted in the absence of any commercial or financial relationships that could be construed as a potential conflict of interest.

## Publisher’s Note

All claims expressed in this article are solely those of the authors and do not necessarily represent those of their affiliated organizations, or those of the publisher, the editors and the reviewers. Any product that may be evaluated in this article, or claim that may be made by its manufacturer, is not guaranteed or endorsed by the publisher.

## Supplementary Material

Supplementary Figure 1 Flow cytometry gating strategy to identify and characterize T lymphoblasts. Blasts were initially identified based on CD34 versus CD38 plots as CD34-positive and CD38-dim events, which were found to express CD45-dim and have low side scatter **(A–D)**, first and second columns). In a T-cell specific flow cytometry tube, blasts were identified based on CD7, CD4 and CD45 as CD7-bright, CD4-negative and CD45-dim events based on flow cytometry findings from A-D €. Finally, in a flow cytometry tube assessing cytoplasmic MPO, CD79a, CD22, CD3 and TdT, blasts were identified based on CD45 versus CD34, as CD34-positive and CD45-dim **(F)**. Each row represents a distinct flow cytometry tube run. The first column in each row is the two-by-two plot used to identify the blast population, and the remaining plots are gated on that given blast population **(A–D, F)** except in the T-cell specific tube which additionally used the CD45 vs SSC plot **(E**, second column) to exclude CD45-bright events to identify blasts. Starred plots served as the presented blast population in **Figure 1B** (red population).

Supplementary Figure 2 Gating strategy to identify and characterize plasmacytoid dendrtic cells (pDCs). pDCs were initially identified based by their characteristic immunophenotype of CD45-moderate, CD123-bright and HLA-DR-bright surface expression (**A**, first and second columns). Based on the markers in this flow cytometry tube, pDCs additionally expressed CD4 and CD38, and were negative for CD13 and other monocytic markers **(A)**. A subsequent flow cytometry tube identified pDCs based on HLA-DR, CD13 and CD45 as HLA-DR-bright and CD13-negative and CD45-moderate **(B)**. In the next flow cytometry tube pDCs were identified based on CD4, CD7 and CD45 as CD4-positive, CD7-moderate and CD45-moderate (**C**, first and second columns). Based on results from A-C, in the subsequent tube, pDCs were identified based on CD7, CD33 and CD45, as CD7-moderate, CD33-moderate, CD45-moderate (**D**, first and second columns). In the next flow cytometry tube pDCs were identified based on CD38, CD34 and CD45 as CD38-positive, CD34-negative and CD45-moderate **(E)**. Finally, in a flow cytometry tube assessing cytoplasmic MPO, CD79a, CD22, CD3 and TdT, pDCs were identified based on CD45 versus CD34, as CD34-negative and CD45-moderate **(F)**. Each row represents a distinct flow cytometry tube run. The first two columns in each row are the two-by-two plots used to identify the plasmacytoid dendritic cell population, and the remaining plots are gated on that given pDC population. Starred plots served as the presented pDC population in **Figure 1B** (blue population).

Supplementary Figure 3 TdT immunostain reactivity. TdT immunostain of bone marrow core biopsy of case report specimen (400X magnification) showing dark brown nuclear staining of TdT-positive lymphoblasts in clusters with low-level background reactivity in pDCs and other non-blast cells **(A)**. TdT immunostain of thymus (400X magnification) showing dark brown nuclear staining of TdT-positive cortical thymocytes with low-level background reactivity in TdT-negative medullary thymocytes.
