# Supplementary figures and images for: Case Report: Mature Plasmacytoid Dendritic Cell Proliferation Associated With a Lymphoid Neoplasm
Source: Front Oncol. 2022 Jul 6;12:903113. doi: 10.3389/fonc.2022.903113 (PMC9296782; doi:10.3389/fonc.2022.903113)

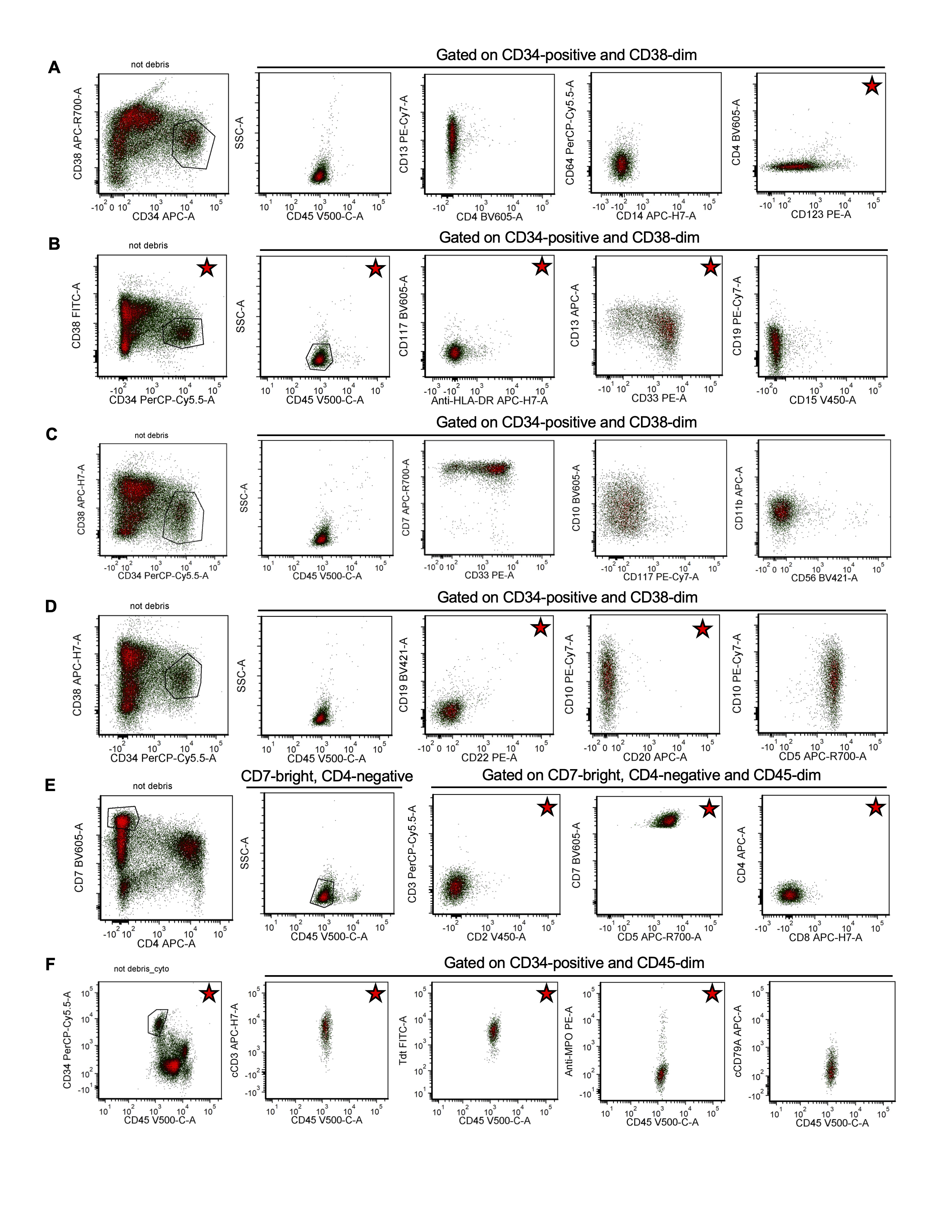

Supplement: Supplementary Figure 1 — Flow cytometry gating strategy to identify and characterize T lymphoblasts. Blasts were initially identified based on CD34 versus CD38 plots as CD34-positive and CD38-dim events, which were found to express CD45-dim and have low side scatter (A–D), first and second columns). In a T-cell specific flow cytometry tube, blasts were identified based on CD7, CD4 and CD45 as CD7-bright, CD4-negative and CD45-dim events based on flow cytometry findings from A-D €. Finally, in a flow cytometry tube assessing cytoplasmic MPO, CD79a, CD22, CD3 and TdT, blasts were identified based on CD45 versus CD34, as CD34-positive and CD45-dim (F). Each row represents a distinct flow cytometry tube run. The first column in each row is the two-by-two plot used to identify the blast population, and the remaining plots are gated on that given blast population (A–D, F) except in the T-cell specific tube which additionally used the CD45 vs SSC plot (E, second column) to exclude CD45-bright events to identify blasts. Starred plots served as the presented blast population in Figure 1B (red population). [file Image_1.tiff]

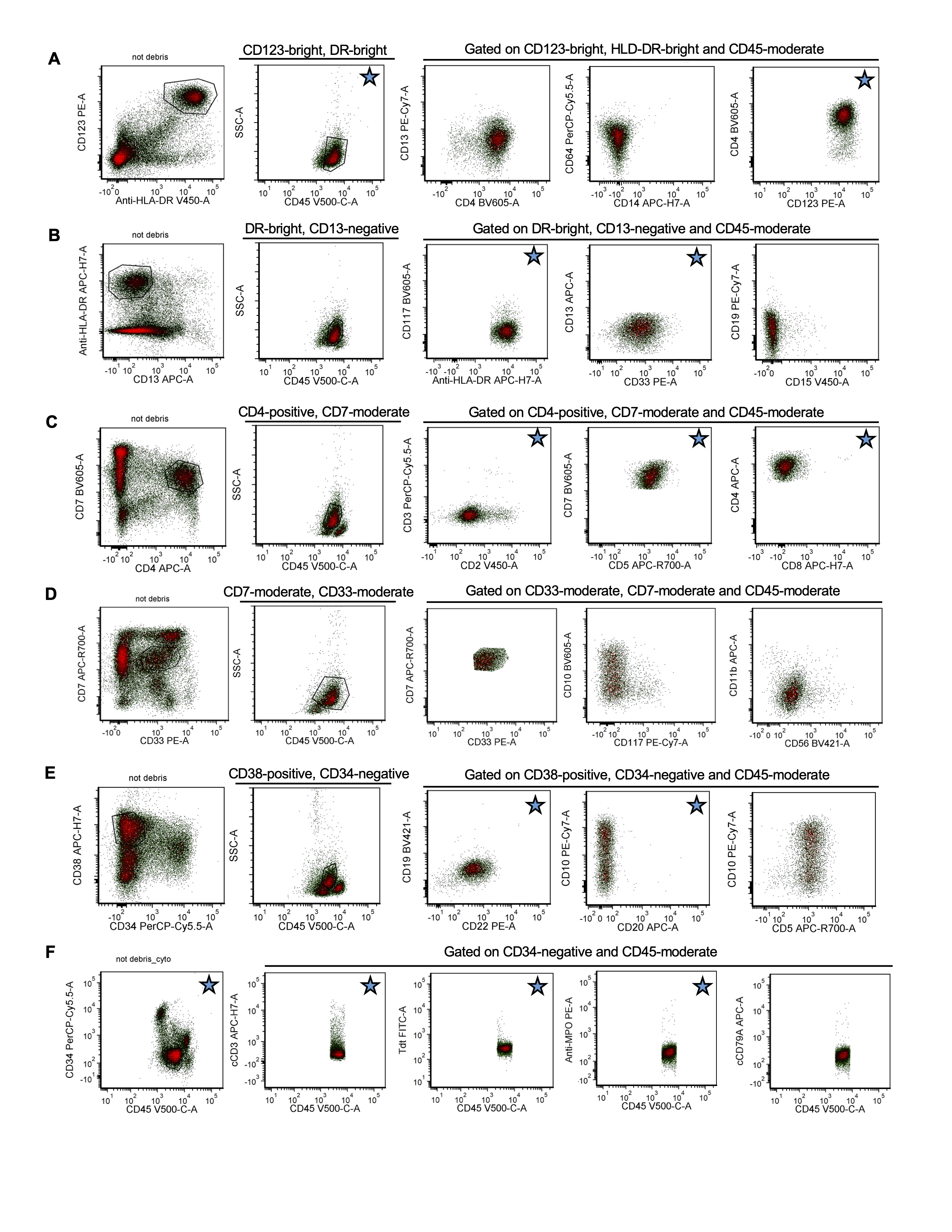

Supplement: Supplementary Figure 2 — Gating strategy to identify and characterize plasmacytoid dendrtic cells (pDCs). pDCs were initially identified based by their characteristic immunophenotype of CD45-moderate, CD123-bright and HLA-DR-bright surface expression (A, first and second columns). Based on the markers in this flow cytometry tube, pDCs additionally expressed CD4 and CD38, and were negative for CD13 and other monocytic markers (A). A subsequent flow cytometry tube identified pDCs based on HLA-DR, CD13 and CD45 as HLA-DR-bright and CD13-negative and CD45-moderate (B). In the next flow cytometry tube pDCs were identified based on CD4, CD7 and CD45 as CD4-positive, CD7-moderate and CD45-moderate (C, first and second columns). Based on results from A-C, in the subsequent tube, pDCs were identified based on CD7, CD33 and CD45, as CD7-moderate, CD33-moderate, CD45-moderate (D, first and second columns). In the next flow cytometry tube pDCs were identified based on CD38, CD34 and CD45 as CD38-positive, CD34-negative and CD45-moderate (E). Finally, in a flow cytometry tube assessing cytoplasmic MPO, CD79a, CD22, CD3 and TdT, pDCs were identified based on CD45 versus CD34, as CD34-negative and CD45-moderate (F). Each row represents a distinct flow cytometry tube run. The first two columns in each row are the two-by-two plots used to identify the plasmacytoid dendritic cell population, and the remaining plots are gated on that given pDC population. Starred plots served as the presented pDC population in Figure 1B (blue population). [file Image_2.tiff]

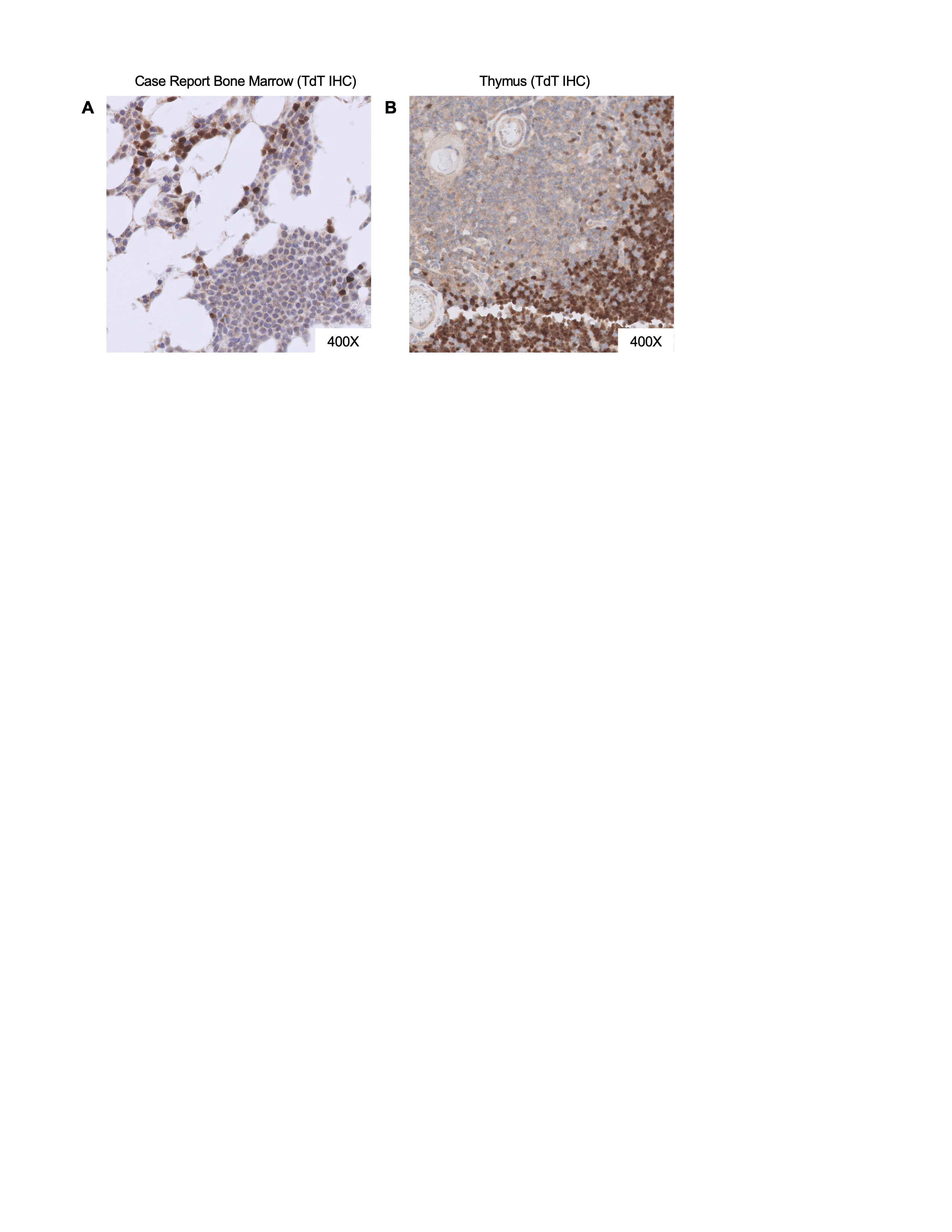

Supplement: Supplementary Figure 3 — TdT immunostain reactivity. TdT immunostain of bone marrow core biopsy of case report specimen (400X magnification) showing dark brown nuclear staining of TdT-positive lymphoblasts in clusters with low-level background reactivity in pDCs and other non-blast cells (A). TdT immunostain of thymus (400X magnification) showing dark brown nuclear staining of TdT-positive cortical thymocytes with low-level background reactivity in TdT-negative medullary thymocytes. [file Image_3.tiff]
